# Percutaneous nephrostomy versus antegrade double-J stent placement in the treatment of malignant obstructive uropathy: a cost-effectiveness analysis from the perspective of the Brazilian public health care system

**DOI:** 10.1590/0100-3984.2018.0127

**Published:** 2019

**Authors:** Tiago Kojun Tibana, Renata Motta Grubert, Rômulo Florêncio Tristão Santos, Vinicius Adami Vayego Fornazari, André Alonso Domingos, William Tavares Reis, Edson Marchiori, Thiago Franchi Nunes

**Affiliations:** 1 Hospital Universitário Maria Aparecida Pedrossian da Universidade Federal de Mato Grosso do Sul (HUMAP-UFMS), Campo Grande, MS, Brazil.; 2 Escola Paulista de Medicina da Universidade Federal de São Paulo (EPM-Unifesp), São Paulo, SP, Brazil.; 3 Faculdade de Medicina da Universidade Federal de Mato Grosso do Sul (FM-UFMS), Campo Grande, MS, Brazil.; 4 Universidade Federal do Rio de Janeiro (UFRJ), Rio de Janeiro, RJ, Brazil.

**Keywords:** Cost-benefit analysis, Radiology, interventional, Nephrostomy, percutaneous, Stents, Análise custo-efetividade, Radiologia intervencionista, Nefrostomia percutânea, Stent

## Abstract

**Objective:**

To compare two percutaneous techniques used in the treatment of malignant obstructive uropathy-antegrade double-J stent placement (JJ stenting) and percutaneous nephrostomy-in terms of their cost-effectiveness, from the perspective of the Brazilian public health care system.

**Materials and Methods:**

In this cost-effectiveness analysis, we employed decision-analytic modeling. We calculated material costs from 2017 factory prices listed by the Brazilian Pharmaceutical Market Regulatory Board (for medications) and published in the journal Revista Simpro (for medical devices). Procedure-related costs were evaluated, as were the rates of technical and clinical success. Those measures were then used as inputs for a cost-effectiveness analysis comparing the two procedures.

**Results:**

The sample comprised 41 patients, of whom 16 underwent antegrade JJ stenting (26 procedures) and 10 underwent percutaneous nephrostomy (15 procedures). Patient records, radiology reports, and expense reports of the interventional radiology department of the public hospital where the study was conducted were analyzed retrospectively. There were no significant complications: one patient had low back pain, and one had a transient retroperitoneal hematoma. The mean procedure time was 24 min, and clinical success (improvement in serum creatinine and resolution of hydronephrosis) was achieved in 97.5% of the cases. The average cost of JJ stenting was significantly lower than was that of percutaneous nephrostomy (US$164.10 vs. US$552.20).

**Conclusion:**

In the absence of any clinical contraindications, antegrade JJ stenting is a suitable alternative to both percutaneous nephrostomy and retrograde stenting in patients with dilated renal collecting systems secondary to malignant ureteral obstruction, providing significant cost savings and high success rates.

## Abstract (pt)

**Objetivo:**

Comparar a relação custo-efetividade de duas técnicas percutâneas utilizadas no tratamento da uropatia obstrutiva maligna - inserção anterógrada de cateter duplo J (JJ) versus nefrostomia percutânea - sob a perspectiva do sistema de saúde pública brasileira.

**Materiais e Métodos:**

Nesta análise de custo-efetividade por modelo analítico de decisão, os custos de material foram calculados a partir dos preços de fábrica de 2017 listados pela Câmara Brasileira de Regulamentação de Medicamentos (para medicamentos) e publicados na Revista Simpro (para dispositivos médicos). Custos relacionados ao procedimento e taxas de sucesso técnico e clínico foram avaliados. Essas medidas foram então usadas como insumos para uma análise de custo-efetividade comparando os dois procedimentos.

**Resultados:**

A amostra foi composta de 41 pacientes, dos quais 16 foram submetidos a 26 procedimentos de inserção anterógrada de JJ e 10 foram submetidos a 15 nefrostomias percutâneas. Registros de pacientes, relatórios de radiologia e relatórios de despesas do serviço de radiologia intervencionista do hospital onde o estudo foi conduzido foram analisados retrospectivamente. Não houve complicações significativas: um paciente apresentou lombalgia e um apresentou hematoma retroperitonial transitório. O tempo médio de procedimento foi 24 minutos e o sucesso clínico (melhora da creatinina sérica e resolução da hidronefrose) foi alcançado em 97,5% dos casos. O custo médio da inserção de JJ (US$ 164.10) foi significativamente menor do que o da nefrostomia percutânea (US$ 552.20).

**Conclusão:**

Na ausência de qualquer contraindicação clínica, a inserção anterógrada de JJ é uma alternativa adequada à nefrostomia percutânea e inserção retrógrada em pacientes com sistema coletor renal dilatado secundário a obstrução maligna, proporcionando economia significativa e altas taxas de sucesso.

## INTRODUCTION

There is a growing demand for interventional radiology in the management of upper urinary tract obstruction secondary to unilateral or bilateral ureteral pathology, especially when attempts at retrograde ureteral stenting fail in cases of malignant obstruction of the distal ureter or when retrograde ureteral stenting is contraindicated (e.g., when ureteral obstruction is accompanied by gram-negative bacterial sepsis or renal failure). Traditionally, this has been addressed with a two-stage approach-percutaneous nephrostomy followed, after a suitable interval, by antegrade stent placement. However, with increasing expertise and advances in technology, radiologists are adopting a single-stage approach in many cases.

There are no clear guidelines regarding the optimal method for urinary decompression in the setting of ureteral obstruction. Within this context, the present study was designed to compare the overall success rates and cost-effectiveness of two techniques-antegrade ureteral stenting and percutaneous nephrostomy-in patients with malignant obstructive nephropathy that is refractory to conventional (retrograde) stenting, from the perspective of the publicly funded Brazilian Unified Health Care System.

## MATERIALS AND METHODS

### Study design

Institutional review board approval was obtained prior to the start of this retrospective study. We selected patients presenting with urinary tract obstruction secondary to malignancy from January 2012 to August 2018. A total of 378 patients underwent cystoscopy with retrograde double-J stent placement (JJ stenting) by a urologist. In 72 patients (19% of the cases), retrograde passage of a JJ stent was impossible. Those patients were referred to the interventional radiology department.

From January 2012 to December 2016, the only technique used at the study facility in cases of urinary tract obstruction refractory to retrograde JJ stenting was percutaneous nephrostomy. In December 2016, the percutaneous antegrade JJ stenting technique was implemented as an alternative for this purpose in cases with no signs or symptoms of sepsis.

### Patient selection

This was a retrospective study (using data from patient records and radiology reports) of 72 patients with obstructive hydronephrosis due to neoplasia. In all of the patients, the condition was refractory to retrograde ureteral stent placement. The patients were stratified into two groups according to the treatment received. Patients who presented with sepsis and underwent primary percutaneous nephrostomy (*n* = 20) were excluded, as were those who underwent percutaneous nephrostomy followed by internalization of the JJ stent (*n* = 11). Therefore, the final sample comprised 41 patients (Table 1). The stenting procedure was performed only if there was no suspicion of sepsis and the patient was hemodynamically stable.

**Table 1:** Indications for the two percutaneous procedures compared.

	Percutaneous JJ stenting (*n* = 26)			Percutaneous nephrostomy (*n* = 15)		
	Indication	*n* (%)		Indication	*n* (%)	
	Bladder cancer	7 (27)		Uterine cancer	5 (33)	
	Uterine cancer	6 (23)		Bladder cancer	5 (33)	
	Metastatic colorectal cancer	4 (15)		Prostate adenocarcinoma	3 (20)	
	Adenocarcinoma of the prostate	3 (12)		Metastatic colorectal cancer	1 (7)	
	Sarcoma of the prostate	3 (12)		Ovarian cancer	1 (7)	
	Colorectal adenocarcinoma	2 (7)				
	Retroperitoneal neuroendocrine tumor	1 (4)				

Prior to December 2016 at our institution, all cases of obstructive uropathy that were refractory to retrograde JJ stenting or in which such stenting was not feasible were treated with percutaneous nephrostomy. Thereafter, because there were improvements in the technique, we opted for anterograde JJ stenting in such cases. For cases of sepsis, hemodynamic instability, or infeasibility of anterograde JJ stenting (stenosis > 5 cm), we opted for percutaneous nephrostomy. The severity and chronicity of obstruction were not considered exclusion criteria. Intravenous antibiotics were given to all patients.

### Percutaneous nephrostomy technique

Prior to percutaneous nephrostomy, ultrasound was performed to ascertain the nature and location of the obstruction. The minimum dilation of the renal pelvis was 20 mm. In all cases, a 10F pigtail catheter was used. A 22G Chiba needle was inserted into the collecting system with a posterolateral approach, through a renal calyx, under ultrasound and fluoroscopic guidance. Once the needle was in the collecting system, urine was aspirated for microbiological analysis, contrast was instilled to identify the anatomy, and a hydrophilic guidewire was passed through the proximal ureter to ensure access. This wire was then replaced with a stiff guidewire (Amplatz SuperStiff; Boston Scientific, Natick, MA, USA). The tract was dilated to 8F and then to 10F. A nephrostomy tube was then placed in the desired position and connected to an external drainage bag.

### Percutaneous antegrade stenting technique

**Figure 1:**
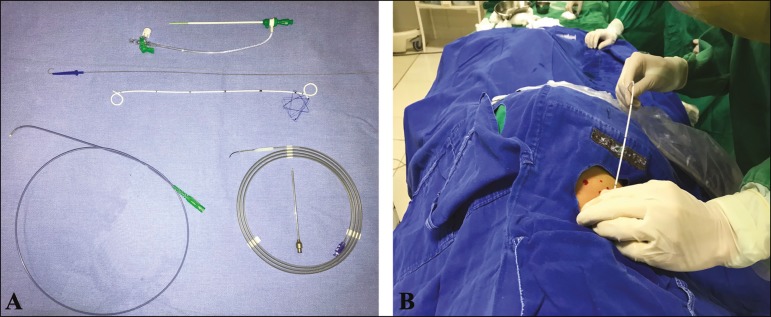
Materials and techniques used in percutaneous JJ stenting (**A** and **B**, respectively).

**Figure 2:**
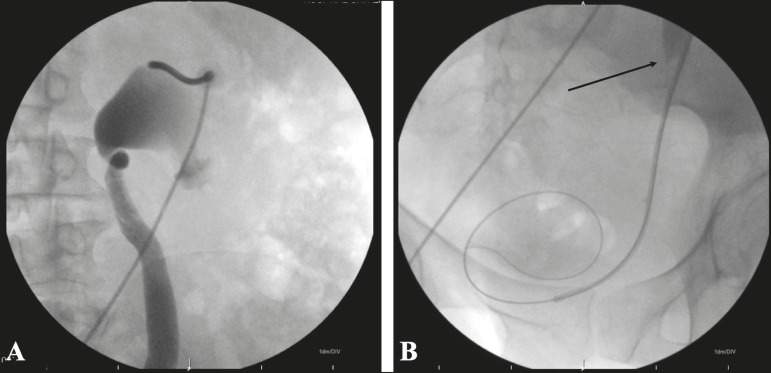
**A**: Fluoroscopy showing puncture of the renal calyx, together with pyelography showing the anatomy of the collecting system. **B**: Transposition of the stenosis (arrow) with a hydrophilic guidewire and catheter inserted into the bladder.

**Figure 3:**
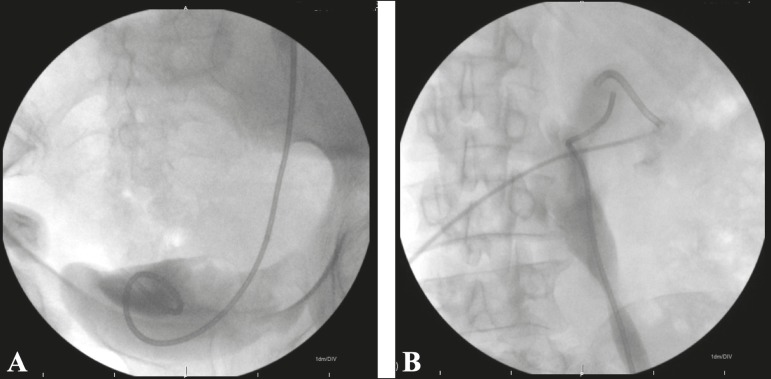
Fluoroscopy showing the distal and proximal ends (**A** and **B**, respectively) of a well-positioned JJ stent.

Percutaneous access (Figure 1) to the collecting system was achieved under ultrasound and fluoroscopic guidance, an 18G echogenic needle being used in order to allow adequate visualization of the advancement of the needle from the skin to the renal calyx. Puncture was performed preferably through the middle calyx, which provides easier access to the ureteropelvic junction, or through a calyx in the lower pole, oriented laterally, which provides a safe and relatively avascular route, the objective being to minimize complications such as bleeding and pneumothorax. Antegrade pyelography (Figure 2A) was performed with injection of iodinated contrast medium and fluoroscopic visualization of the anatomy of the collecting system. Once access had been established, a hydrophilic guidewire and 5F diagnostic catheter were advanced, under fluoroscopy, from the collecting system to the bladder (Figure 2B). A 7F × 45 cm introducer sheath was then put in place, after which the guidewire and 5F catheter were withdrawn. The JJ stent was then passed through the introducer sheath, with the aid of a J-tip polytetrafluoroethylene-coated guidewire (Figure 3). Plain films of the abdomen were obtained 12-72 h after the procedure to visualize the position of the catheter and to assess excretion of the administered contrast.

### Technical and therapeutic success

Technical success of the procedure was defined as maintenance of urinary tract patency and reduction of the severity of hydronephrosis, as determined by imaging (ultrasound or computed tomography). Clinical success was defined as relief of pain and improvement of renal function (resolution of hydronephrosis), with a reduction in the blood levels of nitrogenous waste products (improvement in serum creatinine).

### Follow-up

Thirty days after the percutaneous procedure, patients returned to the interventional radiology clinic for assessment of stent patency, renal function tests (urea and creatinine), a complete blood count, and renal ultrasound. After that assessment, patients scheduled an appointment with the outpatient urology clinic for retrograde JJ stent replacement, which, at our facility, is routinely performed 30 days after antegrade placement. Complications were classified as major or minor, according to the criteria proposed by Goldberg et al.^(^^1^^)^.

### Cost data

The costs of medicines were calculated from factory prices listed by the Pharmaceutical Market Regulatory Board in 2018, whereas the costs of medical devices were obtained from the 2017 volume of the journal **Revista Simpro**. In both cases, the 18% Brazilian tax on the circulation of goods and services was taken into consideration. Human resource costs were not included in the present analysis, because such resources do not apply to our perspective of evaluation.

### Statistical analysis

Data were entered into an Excel spreadsheet and exported to the SPSS Statistics software package, version 20.0 (IBM Corp., Armonk, NY, USA) for statistical analysis. Categorical variables were described as absolute and relative frequencies, whereas quantitative variables were described as mean and standard deviation-when symmetrically distributed-or as mean, median, standard deviation, and interquartile range-when asymmetrically distributed.

Categorical variables were assessed with Fisher’s exact test or the chi-square test. Adjusted residuals analysis was used in order to detect categories with a higher-than-expected frequency. The normality of distribution of the quantitative variables was evaluated by the Kolmogorov-Smirnov test. Symmetrically distributed quantitative variables were compared between groups by Student’s *t*-tests for independent samples, whereas those with an asymmetric distribution were compared by the Mann-Whitney *U* test. The significance level was set at 5% for all comparisons.

## RESULTS

Data were collected from 41 patients, of whom 18 (43.9%) were female and 23 (56.1%) were male. Fifteen patients were treated with percutaneous nephrostomy, and 26 were treated with antegrade JJ placement. In patients with severe hydronephrosis who underwent anterograde insertion, the extent of stenosis was < 5 cm. The mean patient age was 65.6 ± 9.5 years. There was no statistically significant difference between the two groups in terms of the in-patient profiles at baseline (data not shown).

Table 2 shows a comparison of data on the procedures and clinical features of each group. Most patients in the percutaneous JJ stenting group received a 6F stent, whereas all of those in the percutaneous nephrostomy group received a 10F pigtail catheter. Half of the patients in the percutaneous JJ stenting group were discharged less than 12 h after the procedure, compared with only 20% of those in the percutaneous nephrostomy group. The mean cost of antegrade percutaneous JJ stenting was US$164.10 ± 58.40, compared with US$552.20 ± 0.90 for percutaneous nephrostomy, a statistically significant difference (*p* < 0.001; Mann-Whitney *U* test).

**Table 2:** Comparison between the two percutaneous procedures.

Variable	Percutaneous JJ stenting (*n* = 26)	Percutaneous nephrostomy (*n* = 15)	*p*
Severity of hydronephrosis, n (%)			0.278*
Mild	1 (3.8)	—	
Moderate	3 (11.5)	—	
Severe	22 (84.6)	15 (100.0)	
Post-procedure acute kidney injury, *n* (%)	13 (50.0)	6 (40.0)	0.769*
Duration of the procedure (min), mean ± SD	23.3 ± 9.7	20.4 ± 3.3	0.179†
Complications, *n* (%)			0.543*
None	25 (96.2)	13 (86.7)	
Minor	1 (3.8)	2 (13.3)	
Major	—	—	
Access, *n* (%)			0.854*
Right	10 (38.5)	7 (46.7)	
Left	16 (61.5)	8 (53.3)	
JJ stent diameter, *n* (%)			< 0.001*
4F	3 (11.5)	—	
6F	23 (88.5)	—	
10F	—	15 (100.0)	
Time to discharge, *n* (%)			0.030*
< 12 h	13 (50.0)	3 (20.0)	
12-24 h	10 (38.5)	12 (80.0)	
24-48 h	3 (11.5)	—	
> 48 h	—	—	
Drain migration, *n* (%)	1 (3.8)	3 (20.0)	0.130*

SD, standard deviation.

* Chi-square test or Fisher’s exact test.

† Student’s *t*-test for independent samples.

## DISCUSSION

Drainage of the urinary tract can be performed via several techniques and devices, including retrograde cystoscopy, antegrade percutaneous insertion of a JJ stent, and percutaneous nephrostomy. The drawbacks of external drainage systems include the risk of infection and displacement, as well as the discomfort of an external catheter. The objective of therapy is to achieve adequate drainage of the urinary tract for the maintenance of renal function; in this context, antegrade JJ stenting has become an important interventional radiology procedure^(^^2^^)^.

Previous studies^(^^3^^,^^4^^)^ have shown that the incidence of retrograde stenting failure is significantly higher in cases of malignant extrinsic compression, and that in most cases of bladder tumor or prostate carcinoma, percutaneous nephrostomy is preferable, because stenting would not be possible due to tumor encroachment into the ureteral orifices. Ku et al.^(^^5^^)^, Chang et al.^(^^6^^)^, and Nariculam et al.^(^^7^^)^ also found percutaneous nephrostomy to be the best option for temporary urinary diversion in extrinsic obstructive uropathy due to advanced-stage malignant neoplasms.

In the present study, antegrade JJ stenting was performed successfully in 97.5% of the patients, comparable to the 94.2% reported by Memon et al.^(^^8^^)^. In one of the cases evaluated in the present study, in which antegrade JJ stenting was unsuccessful (the stent could not be placed, because there was extensive ureteral involvement), we chose to perform percutaneous nephrostomy. We found that percutaneous nephrostomy was performed successfully in 100% of the patients evaluated in our study, whereas Naeem et al.^(^^9^^)^ and Wah et al.^(^^10^^)^ reported success rates of 96.1% and 98.0%, respectively.

In the present study, the most common complication was self-limited perirenal hematoma, which occurred in two cases in the percutaneous nephrostomy group and in one case in the percutaneous JJ stenting group. Naeem et al.^(^^9^^)^, Jalbani et al.^(^^11^^)^, and Romero et al.^(^^12^^)^ observed that complication in 4.0%, 5.0%, and 3.5% of their patients, respectively. Painful irritation of the bladder trigone was not reported in our patient sample, whereas that complication occurred in 10% and 9% of the patients evaluated by Shao et al.^(^^13^^)^ and Memon et al.^(^^8^^)^, respectively. In addition, we did not observe any cases of post-stenting septicemia, the incidence of which was 10.2% in the study conducted by Arshad et al.^(^^14^^)^. Nephrostomy drain migration was observed in two (13%) of the patients in our sample. In previous studies, the reported incidence of that complication was 4-37%^(^^7^^,^^9^^,^^11^^)^. Memon et al.^(^^8^^)^ and Arshad et al.^(^^14^^)^ observed JJ stent encrustation in 17.5% and 2.0% of their patients, respectively, as well as JJ stent migration in 11.7% and 16.3%, respectively. In our study, JJ stent encrustation was observed in three cases (11%) and JJ stent migration was observed in one case (3%). In our patients, stents were left in place for a maximum of three months.

The evaluation of urinary tract obstruction and ureterolithiasis by imaging methods has been the subject of a series of recent publications in the radiology literature of Brazil^(^^15^^-^^18^^)^. The present study provides evidence of the value of applying methods of cost-effectiveness analysis to interventional radiology. Although interventional procedures may have a high initial cost, because of the equipment needed or the time consumed, as for all minimally invasive techniques, these costs are expected to be recovered through reductions in morbidity and bed occupancy. Cost analyses can demonstrate this objectively. Cost-effectiveness analysis is particularly suitable for interventional radiology, because it provides a means of comparing strategies with the same unit of benefit or effectiveness. The measure of efficacy in the present study was successful ureteral drainage. A similar objective measure can be identified for most interventional procedures.

Hyams et al.^(^^19^^)^ set out to compare the preferred methods for ureteral drainage in patients with malignant urinary tract obstruction. The authors found that there was significant disagreement between urologists and oncologists regarding the management of hypothetical clinical vignettes. For example, oncologists were more likely to recommend percutaneous nephrostomy as the next logical step after stent failure in unilateral obstruction (79% vs. 62%). Conversely, urologists were more likely to suggest stent manipulation, including increased diameter, stent replacement, internalization, etc. (37% vs. 17%). In addition, the perception of complications differed between the two groups. A greater proportion of urologists considered nephrostomy tube migration to be the greatest risk after percutaneous nephrostomy (48% vs. 18%), whereas a greater proportion of medical oncologists considered it to be infection (40% vs. 8%). Regarding ureteral stent placement, urologists were more concerned with the impact on quality of life (65% vs. 13%), and oncologists were again primarily concerned with the risk of infection (43% vs. 3%). It is noteworthy that urologists and oncologists alike agreed that ureteral stents increased patient comfort (87% and 93%, respectively) and improved quality of life (95% and 93%, respectively).

Our study has some limitations, not the least of which is the small sample size. Another limitation is that cost assessment was restricted to the materials used in the competing techniques. A broader cost analysis, including staff fees, medication costs, operating room time, and admission-related expenditures, might provide a better picture of the overall costs.

In conclusion, in the absence of any clinical contraindications, antegrade percutaneous JJ stenting is a suitable alternative to both percutaneous nephrostomy and retrograde stenting in patients with dilated renal collecting systems secondary to malignant ureteral obstruction. In comparison with percutaneous nephrostomy, antegrade percutaneous JJ stenting provides significant cost savings while maintaining high success rates.
